# Dietary Inflammatory Index of Northern Mexican Indigenous Adults and Its Association with Obesity: Cross-Sectional Study

**DOI:** 10.3390/nu18020249

**Published:** 2026-01-13

**Authors:** José M. Moreno-Abril, Mónica D. Zuercher, Silvia Y. Moya-Camarena, Heliodoro Alemán-Mateo, Araceli Serna-Gutiérrez, René Urquidez-Romero, Ana C. Gallegos-Aguilar, Julián Esparza-Romero

**Affiliations:** 1 Obesity and Diabetes Research Unit, Nutrition Coordination, Research Center for Food and Development (CIAD), A.C., Carretera Gustavo Enrique Astiazarán Rosas, No. 46 Col. La Victoria, Hermosillo 83304, Sonora, Mexico; 2 Nutrition Policy Institute, University of California Division of Agriculture and Natural Resources, Oakland, CA 94607, USA; 3 Molecular Nutrition Laboratory, Nutrition Coordination, Research Center for Food and Development (CIAD), A.C., Hermosillo 83304, Sonora, Mexico; 4 Laboratory of Body Composition and Functionality, Nutrition Coordination, Research Center for Food and Development (CIAD), A.C., Hermosillo 83304, Sonora, Mexico; 5 Sociocultural Department, Technological Institute of Sonora, Ciudad Obregón 85137, Sonora, Mexico; 6 Department of Health Sciences, Institute of Biomedical Science, Autonomous University of Ciudad Júarez, Ciudad Júarez 32310, Chihuahua, Mexico

**Keywords:** obesity, abdominal obesity, dietary inflammatory index, pro-inflammatory diet, Indigenous population, nutritional transition, traditional diet

## Abstract

**Background/Objectives**: Given the high prevalence of obesity and abdominal obesity in Indigenous adults from Sonora (IAS) and its strong association with diet, this study evaluates the association of dietary inflammatory index (DII) with obesity and abdominal obesity and its indicators, such as body mass index (BMI) and waist circumference (WC), respectively. **Methods**: This cross-sectional study included data from 559 adults across two Indigenous populations (Seris and Yaquis) collected in two separate studies. Obesity and abdominal obesity were classified according to the definitions established by the World Health Organization and the International Diabetes Federation. The DII was calculated with data from population-specific food frequency questionnaires. Multiple linear regression was used to assess the association between the DII variable (expressed as both numeric and categorical) and BMI and WC, separately; multiple logistic regression was used to evaluate the association between obesity and abdominal obesity. **Results**: The prevalence of obesity and abdominal obesity was 34.1% and 78.2%, respectively. There was a positive association between the DII and BMI (DII as numeric: β = 0.53, *p* = 0.001; tertile3 of DII vs. tertile1: β = 1.86, *p* = 0.001) and WC (DII as numeric: β = 1.15, *p* = 0.002; tertile3 of DII vs. tertile1: β = 3.81, *p* = 0.005). Similar results were found for both types of obesity. **Conclusions**: Higher DII scores were associated with increased obesity indicators (BMI and WC) and a higher risk of obesity and abdominal obesity in IAS. Promoting anti-inflammatory diets represents a feasible approach for preventing non-communicable diseases.

## 1. Introduction

Obesity is recognized as one of the main pandemics, with associated comorbidities that significantly contribute to the increasing premature mortality across different regions of the world [1,2]. Over the last three decades, the global prevalence of obesity has doubled in women (from 10.2% to 20.8%) and nearly tripled in men (from 5.8% to 14.8%) [1]. In Mexico, the National Health and Nutrition Survey 2022 (ENSANUT for its Spanish acronym, 2022) reported an obesity prevalence of 36.9% in adults, being higher in women (41.0%) than in men (32.3%), and an abdominal obesity prevalence even higher (81.0%), more frequent among women (88.0%) than men (74.0%) [3].

Among Northern Mexican Indigenous groups, obesity is also a major health concern. A study in the Seri (Comcáac) adult population reported that 26.0% of the population had obesity and 74.0% had abdominal obesity [4]. In another study of Yaqui (Yoeme) adults [5], the prevalence of obesity and abdominal obesity was 35.0% and 76.0%, respectively. In Yaquis, higher time spent in vigorous physical activity and greater adherence to a “prudent” dietary pattern were associated with lower body mass index (BMI). In contrast, a higher modernity index or having a greater number of household goods and services was associated with a higher BMI. The “prudent” dietary pattern was characterized by a high intake of traditional dishes, vegetables, fruits, and low-fat dairy products [5]. These findings reflect the ongoing nutritional transition among Indigenous peoples [6]. However, individuals who maintain a traditional diet have a lower risk of developing non-communicable diseases (NCDs) [5,7].

A Westernized dietary pattern, in contrast, is one of the most critical factors associated with the development of obesity and NCDs [8]. Low-grade chronic inflammation induced by diet is considered a key factor in explaining these associations [9]. Evidence shows how pro-inflammatory diets may contribute to obesity, particularly [10,11,12]. Diets high in fat and refined sugar promote intestinal dysbiosis and subsequent systemic endotoxemia [9,12,13,14]. This condition increases pro-inflammatory cytokines, which stimulate insulin overproduction [9]. As an anabolic hormone, insulin promotes triglyceride storage in adipose tissue, allowing for its expansion and promoting obesity [15].

As a non-invasive instrument, the Dietary Inflammatory Index (DII) was proposed to assess the inflammatory or anti-inflammatory potential of an individual’s dietary intake [16]. The DII, developed by Shivappa and colleagues [16], comprises 45 dietary parameters (energy, nutrients, and foods) and utilizes a scoring system ranging from −7.98 to 8.90. More negative DII scores represent a more anti-inflammatory diet, while more positive values indicate a pro-inflammatory diet [16].

High DII scores have been consistently associated with NCDs [17], including diabetes [18], metabolic syndrome [19,20], cardiovascular disease [18,21], neurodegenerative diseases [22,23], and cancer [24,25]. Other studies have also reported associations with obesity [26,27], abdominal obesity [28,29], and increases in obesity indicators, such as body mass index (BMI) [30,31] and waist circumference (WC) [30,32]. However, some studies have not found significant associations [19,33]. Although it has been documented that Indigenous populations are undergoing a nutritional transition, no studies to date have evaluated the association between DII and obesity or other NCDs in these populations.

Given this context, the objective of the present study is to assess the association between DII scores and measures of obesity, as well as their prevalence among Indigenous adults from Sonora (IAS).

## 2. Materials and Methods

### 2.1. Study Design

This work is a secondary analysis based on two cross-sectional studies conducted among two Indigenous adult populations in Sonora: the Seris (2015) [7] and the Yaquis (2017) [5]. Both studies were approved by the Institutional Ethics Committee (approval codes CE/005/2013 and CE/007/2016).

### 2.2. Participants

For this secondary analysis, 578 adults (aged 20 years or older) of both sexes were included: 227 Seri and 351 Yaqui participants. Pregnant or breastfeeding women, bedridden individuals, and those with a history of psychiatric disorders or mobility impairments were excluded from both studies. Individuals whose dietary data demonstrated inconsistencies were excluded from the analysis.

The sampling procedures for both studies have been published elsewhere [5,7]; a brief summary is provided below. In the Seri study, all adults residing in the localities of Punta Chueca and El Desemboque were invited to participate in [7]. In the Yaqui study, due to the larger population size, a multistage probabilistic sampling was conducted, inviting 351 adults from the traditional communities of Pótam, Vícam Estación, Vícam Pueblo, Loma de Guamúchil, Loma de Bácum, Tórim, Ráhum, Belem, and Casas Blancas [5,34].

### 2.3. Anthropometric Measurements and Obesity Definitions

In both studies, trained staff were assisted by local health promoters to gain a deeper understanding of the communities. Anthropometric measurements included height, body weight, and WC. Weight in the Seri population was measured using a digital scale with a capacity of 150 kg ± 50 g (OHAUS Defender™ 3000, Parsippany, NJ, USA) and a scale with a capacity of 200 kg ± 100 g (SECA™ 813, Hamburg, Germany) in the Yaqui population. Height was measured using a portable stadiometer (HOLTAIN™, Dyfed, UK) in the Seri population and a Seca 213 portable stadiometer (SECA™, Hamburg, Germany) in the Yaqui population. WC was measured using a fiberglass tape (Lafayette Instruments Company™, Lafayette, IN, USA) for Seri and a Gulick™ fiberglass tape for Yaqui. In the Yaqui population, equipment calibration and measurements were performed by trained personnel using the methodology of the International Society for the Advancement of Kinanthropometry [35]. In the Seri population, measurements were taken according to the protocol outlined by Roblez-Ordaz et al. (2018) [7].

BMI was calculated by weight (kg) divided by the square of height (m^2^). According to the WHO criteria [36], overweight was defined as a BMI ≥ 25 kg/m^2^, and obesity as a BMI ≥ 30 kg/m^2^. Abdominal obesity was classified using the International Diabetes Federation cutoff points for Ethnic South and Central Americans: WC ≥ 80 cm in women and ≥90 cm in men [37].

### 2.4. Dietary Assessment and DII Calculation

Dietary intake was assessed using food frequency questionnaires (FFQs) designed to represent the average annual diet for each population. The Seri FFQ included 88 items, and the Yaqui FFQ included 123 [38,39]. Both FFQs included traditional dishes and seasonal food of each population. Participants were asked about the frequency of consumption (daily, weekly, monthly, or rarely) and portion size (small, medium, or large). Daily intake (g/day) was estimated for each food item, representing the previous 12 months.

The dietary database was cleaned following the methodology used in ENSANUT 2018–19 [40]. Nutrient and energy intakes were estimated using the Food Processor software, version 11 (Trustwell™, Oak Brook, IL, USA). Nutrient intake was adjusted for energy using the residual regression method. Nutrient intake was adjusted by energy using the residual method. This approach uses a simple linear regression model for each nutrient, with the nutrient intake as the dependent variable and energy intake as the independent variable. The residuals from these models represent the variation in nutrient intake independent of energy. Posteriorly, each residual is added to the overall mean of the respective nutrient to obtain each subject’s energy-adjusted nutrients [41].

The DII was calculated following the methodology proposed by Shivappa and colleagues [16]. The DII was computed using an algorithm that comprised 45 parameters, including energy, nutrients, and food.

A Z-score was created for each available parameter to calculate an individual’s DII. The Z-score was obtained by subtracting the “global mean intake” from the individual’s intake of the parameter and dividing it by the “global standard deviation”. The Z-score was then converted into a percentile (ranging from 0 to 1), which was subsequently transformed into a centered percentile (ranging from −1 to +1). This centered percentile was multiplied by the corresponding parameter’s “inflammatory effect score” to obtain the parameter-specific DII. Finally, the overall DII for each subject was calculated as the sum of all parameter-specific DII values. Negative scores indicate an anti-inflammatory diet, whereas positive scores indicate a pro-inflammatory diet.

For this study, 30 of the 45 original parameters were used: energy, protein, carbohydrates, total fiber, total fat, saturated fat, monounsaturated fat, polyunsaturated fat, trans fat, cholesterol, vitamin A, β-carotene, vitamin B1, B2, B3, B6, B12, vitamin C, vitamin D, vitamin E, folate, iron, magnesium, selenium, zinc, omega-3, omega-6, alcohol, onion (all types), and chili pepper (genus Capsicum).

### 2.5. Potential Confounding Variables

The potential confounding variables evaluated were sex, age, sociodemographic-related variables (including the modernization index), clinical-related history variables, alcohol consumption and smoking, and physical activity. Sociodemographic-related variables were evaluated by a sociodemographic questionnaire [5,7]. Among the sociodemographic-related variables included ethnicity (Yaqui or Seri), type of housing (owned/rented or living with parents/relatives), if participant receives government support (yes or no), if is beneficiary of the Seguro Popular program (public health insurance, yes or no), marital status (single, married, consensual union or divorced/widowed), level of education (primary, secondary or high school or higher), and the household goods and services available (e.g., fan, blender, gas stove, internet, DVD, oven, iron, refrigerator, air conditioning, TV, telephone, radio, washing machine, cell phone, electricity, car, and wood stove). The modernization index was calculated as the sum of household goods and services available giving values ranging from 0 to 17 [42]. Clinical-related history variables were evaluated by clinical history questionnaire and included a previous diagnosis of diabetes (yes or no) or hypertension (yes or no), medical treatment (yes or no), a parental history of diabetes and/or hypertension (yes or no), alcohol consumption (yes or no) and current smoker (yes or no) [5,7]. Physical activity was evaluated using physical activity questionnaires adapted for each population, and the results were reported according to their type (recreational or occupational) and level (sedentary, light, moderate, or vigorous), with the findings expressed in hours per week [43,44].

### 2.6. Statistical Analysis

Overweight and obesity prevalences were compared by sex using ordinal logistic regression, and abdominal obesity prevalences were compared by sex using chi-squared.

To compare IAS with anti-inflammatory diets to those with pro-inflammatory diets, participants were grouped into tertiles based on their DII score: tertile 1 (most anti-inflammatory diets) and tertile 3 (most pro-inflammatory diets). Variables between extreme tertiles were compared using independent *t*-tests for normally distributed variables, Mann–Whitney tests for non-normally distributed variables, and chi-square tests for categorical variables.

The association of the DII with BMI and WC was evaluated using multiple linear regression. BMI and WC were included as dependent variables in separate models. In all models, DII was considered the exposure variable, which was analyzed either as a continuous or as a categorical variable in tertiles (using the first tertile as the reference) in separate models. As a secondary analysis, we examined the association between DII and the prevalence of obesity or abdominal obesity using multiple logistic regression models.

The β coefficient, which is the association measure of multiple linear regression, is interpreted in two ways, depending on how the independent variable is expressed. When the independent variable is categorical (Tertile 3 vs. Tertile 1 of the DII), the β coefficient represents the mean difference in the units of the dependent variables (kg/m^2^ for BMI, or cm for WC) between the persons that consumed the most pro-inflammatory diet (those in tertile 3) versus those with the most anti-inflammatory diet (Tertile 1). On the other hand, when the independent variable is expressed as continuous (such as the DII score), the β coefficient reflects the change in the dependent variable associated with a one-unit increase in the independent variable, and that this effect is the same regardless of the individual’s position on the scale.

Similarly, the odds ratio (OR), which is the association measure of multiple logistic regression, is also interpreted in two ways, is also interpreted in two ways, depending on how the independent variable is expressed. When the independent variable is categorical and is a risk related variable (example: Tertile 3 vs. Tertile 1 of the DII), the OR represents how many times great the likelihood is of having overall obesity or abdominal obesity for the group of people who consume a pro-inflammatory diet (those in Tertile 3) in comparison to those with the most anti-inflammatory diet (Tertile 1). On the other hand, when the independent variable is expressed as continuous (such as the DII score), the OR represents how many times greater the likelihood is of having overall obesity or abdominal obesity associated with a one-unit increase in the independent variable, and that this effect is the same regardless of the individual’s position on the scale.

Assessing the DII both as a categorical and a continuous variable provides complementary information. As a DII categorical, it allows us to compare the lowest versus the highest DII categories; whereas, as a DII continuous, it allows us to evaluate the effect associated with each one-unit increase in the DII score.

All the multiple regression models were developed by first conducting an exploratory analysis of all variables in the database, then assessing the potential association between the corresponding outcome variable and each independent variable, using simple regression (*p* ≤ 0.20) and considering the biological plausibility of the regression coefficient β or the odds ratio. Variables with potential associations were subsequently entered into a Stepwise forward analysis to obtain preliminary significant models (*p* ≤ 0.05). Each of the multiple regression models was evaluated for the presence of effect modification (*p* ≤ 0.10), collinearity (VIF < 10), and for their corresponding regression assumptions. A trend test was used (*p* trend ≤ 0.05) to determine if a dose–response relationship existed between obesity and DII tertiles.

All analyses were performed using STATA 18 BE (StataCorp LLC™, College Station, TX, USA). Statistical significance was considered when the *p*-value was ≤ 0.05.

## 3. Results

Of the 578 participants, 559 were included in this study, with 18 excluded due to inconsistencies in dietary intake data. Among the analyzed subjects, 223 were Seris and 336 were Yaquis.

**Figure 1:**
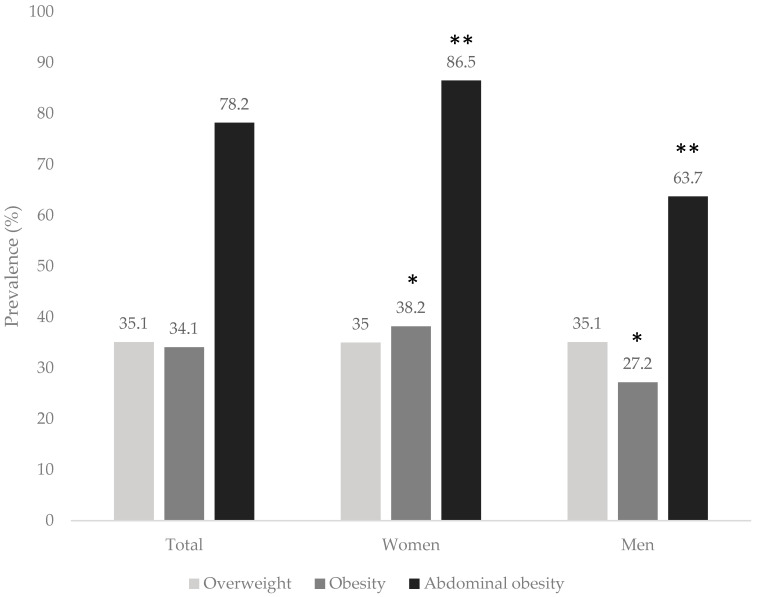
Prevalence of overweight, obesity, and abdominal obesity as a whole and by sex of Indigenous adults in Sonora. * Significant difference in overweight and obesity by sex categories using ordinal logistic regression (*p* = 0.001). ** Significant difference in abdominal obesity using Chi-squared test (*p* = 0.0001).

The prevalence of overweight, obesity, and abdominal obesity in the IAS population was 35.1%, 34.1%, and 78.2%, respectively (Figure 1). Compared with men, women showed a higher prevalence of obesity (women, 38.2% vs. men, 27.2%, *p* = 0.001) and abdominal obesity (women, 86.5% vs. men, 63.7%, *p* = 0.0001). Conversely, overweight prevalence was similar by sex (women, 35.0% vs. men, 35.1%, *p* = 0.852).

The extreme tertile groups of the DII (tertiles 1 and 3) were compared in terms of their anthropometric, sociodemographic, health, and physical activity characteristics, as shown in Table 1. The lowest DII score (the most anti-inflammatory diet) was −4.24, and the highest (the most proinflammatory diet) was 3.73. In the anthropometric measurements, BMI was lower among individuals in tertile 1 (*p* < 0.05), whereas no differences were observed in weight or WC. Regarding sociodemographic information, tertile 1 included older participants, a higher proportion of women, and a greater number of adults who reported using their Indigenous language (*p* < 0.05). Finally, individuals in tertile 1 reported more hours of light, moderate, and vigorous physical activity than those in tertile 3 (*p* < 0.05).

Regarding the DII score contribution of the parameters, the consumption of vitamins, minerals, omega-3 fatty acids, fiber, and foods such as onion and chili peppers distinguished individuals in tertile 1 compared with those in tertile 3, as denoted by a cross (^†^) in Table 2. This anti-inflammatory dietary contribution was characterized by higher intake of β-carotene, vitamin D, vitamin C, magnesium, vitamin A, vitamin B6, onion, zinc, omega-3 fatty acids, fiber, vitamin E, selenium, chili pepper, and niacin in subjects of tertile 1 compared with tertile 3 (*p* < 0.05). Accordingly, lower intake of these parameters brings higher DII scores, which leads to a pro-inflammatory diet in these populations. A significant difference was observed in the parameters’ intakes of energy, omega-3 fatty acids, carbohydrates, iron, monounsaturated fatty acids, and trans fatty acids (indicated by an asterisk, *). However, no significant differences were found between the two tertile groups in the DII scores for energy, omega-6 fatty acids, carbohydrates, riboflavin, iron, monounsaturated fatty acids, trans fats, total fat, saturated fat, and polyunsaturated fatty acids. Conversely, tertile 1 exhibited higher pro-inflammatory scores for protein, thiamine, alcohol, cholesterol, vitamin B12, and folate (*p* < 0.05). Nevertheless, it is anticipated that certain parameter intakes and DII scores may have exhibited a proinflammatory tendency, even within the most anti-inflammatory diet, when compared with those in the third tertile.

The DII was positively associated with both obesity indicators across the different models (Table 3). In the first regression model (Model 1), where the DII was analyzed as a continuous variable, it was found that each one-point increase in the DII scores was associated with a 0.53 kg/m^2^ increase in BMI (β = 0.53, *p* = 0.001, 95% CI: 0.22, 0.85) in the IAS population (Table 3). In the second model (Model 2), where the DII was analyzed in tertiles, individuals in tertile 3 showed a BMI 1.86 kg/m^2^ higher than those in tertile 1 (β = 1.86, *p* = 0.001, 95% CI: 0.72, 3.01). This means that the average BMI among individuals with the most pro-inflammatory diet was 1.86 kg/m^2^ higher than that of those with the most anti-inflammatory diet. In addition, a linear increase in β was observed for each upward change in DII category (*p* for trend = 0.045), indicating a positive dose–response effect. Both models were adjusted for sex, previous diagnosis of hypertension, modernity index, housing type, and vigorous recreational physical activity.

For Model 3, where the association between WC and the DII was analyzed quantitatively (Table 3), it was found that each one-point increase in the DII was associated with a 1.15 cm increase in WC (β = 1.15, *p* = 0.002, 95% CI: 0.41, 1.87). In Model 4, using the DII categorized into tertiles, it was found that individuals in tertile 3 had an average WC 3.81 cm higher (β = 3.81, *p* = 0.005, 95% CI: 1.16, 6.47) compared with those in tertile 1. Like Model 2, WC increased linearly; the mean WC increased progressively from the first to the third tertile (*p* for trend = 0.019), demonstrating a positive dose–response effect. Both models were adjusted for sex, age, modernity index, previous diagnosis of hypertension, at least one parent with diabetes, and vigorous recreational physical activity.

In the secondary analysis, logistic models confirmed a positive association between the DII and both types of obesity (Table 4). Model 1 indicated that the likelihood of obesity increased by 1.25 times with each unit increment in DII scores (OR: 1.25; *p* = 0.001; 95% CI: 1.09, 1.43). A similar effect was observed with abdominal obesity in Model 3 (OR: 1.20; *p* = 0.037; 95% CI: 1.01, 1.42). In Model 2, people who have the most proinflammatory diet (tertile 3) have twice the likelihood of obesity than people who have the most anti-inflammatory diet (OR T3 vs. T1: 2.16; *p* = 0.002; 95% CI: 1.34, 3.45). This model demonstrated that the risk increased with each successive change in DII category (*p* trend = 0.013). In contrast, Model 4 has shown a suggestive trend in the likelihood of abdominal obesity between Tertile 1 and 3 (OR T3 vs. T1: 1.69; *p* = 0.074; 95% CI: 0.95, 2.99). However, people of the tertile 2 had more than twice the likelihood of having abdominal obesity than people from the tertile 1 (OR T2 vs. T1: 2.26; *p* = 0.007; 95% CI: 1.24, 4.10), although not significant (*p* trend = 0.760).

## 4. Discussion

The present study found that Indigenous adults from Sonora (IAS) who consumed a pro-inflammatory diet had increased BMI and WC, as well as a higher prevalence of obesity and abdominal obesity, compared to those who followed an anti-inflammatory diet. Increases in DII category (e.g., from tertile 1 to 2 or from tertile 2 to 3) are associated with progressively higher obesity indicators and greater risk of obesity, demonstrating a precise dose–response effect. Our findings are consistent with previous studies among Mexican and worldwide populations, showing that there is a risk of increased obesity markers with each additional unit of increase in the DII score, regardless of the individual’s position on the DII scale. For example, each 1-point increase in the DII was associated with a 1.15 cm increase in WC, regardless of whether the 1-point increase means moving from a −4 DII score to a −3 DII score or from a 2 DII score to a 3 DII score, the associated effect would still be a 1.15 cm increase in WC in both scenarios. This effect has been documented in various studies involving Mexican and global populations [28,30,31,45,46,47,48]. The mechanisms by which a pro-inflammatory diet increases the risk of obesity-related indicators have been explained by Cani and colleagues [9]. According to their findings, intestinal dysbiosis caused by a potentially pro-inflammatory diet can trigger metabolic endotoxemia, leading to increased production of pro-inflammatory cytokines in the body, elevated insulin secretion, and, consequently, increased fat storage in adipose tissue [9,15,31].

The diet of the IAS group that consumed the most anti-inflammatory diet was characterized by a high intake of anti-inflammatory components, including vitamins, minerals, fiber, omega-3 fatty acids, as well as onions and chili peppers. This anti-inflammatory dietary pattern consisted of a high consumption of fruits, vegetables, healthy fats, fish, beans, and whole-grain flour, as well as corn tortillas [49]. Multiple studies have shown that the consumption of vegetables, fruits, and fish provides essential dietary components characteristic of an anti-inflammatory diet, including vitamins, minerals, and omega-3 fatty acids [50,51,52]. In a cross-sectional study with a Chinese population, the authors found that those who had the most anti-inflammatory diet consumed greater amounts of polyunsaturated fatty acids, fiber, niacin, thiamine, riboflavin, folic acid, vitamin A, vitamin B6, vitamin B12, vitamin C, vitamin E, iron, magnesium, selenium, and zinc, compared to those with the most pro-inflammatory diet [50]. Similarly, in another cross-sectional study involving a European population (the HELENA project), it was reported that individuals with the most anti-inflammatory diets ate more fruits, vegetables, and fish, while consuming less meat, animal fats, sweetened drinks, and sweetened desserts [51].

The foods incorporated in the most anti-inflammatory diet in the IAS group are similar to those included in the “traditional” or “prudent” dietary patterns (the latter including traditional dishes) reported in two studies conducted in the Seri and Yaqui populations. The adherence to these patterns had a protective effect against NCDs, including obesity [5,7]. Consistent with this, our study found that the people who follow an anti-inflammatory diet tend to preserve more of their cultural heritage, being predominantly older, female, and who speak their native language. Several studies have also noted that acculturation plays a significant role in health outcomes [53,54,55]. Therefore, the potential health benefits associated with traditional dietary patterns may be attributed to their anti-inflammatory properties. Integrating this factor, alongside relevant cultural considerations, into future interventions could potentially enhance health outcomes in the IAS.

Despite existing geographic and cultural differences, the traditional diets of both ethnic groups may exert an anti-inflammatory effect. Regarding the Yaqui group, various authors have pointed out that their diet, practiced for over three centuries, was primarily characterized by the consumption of vegetables, whole grains, legumes, as well as wild fruits and regional plants [5,56]. This diet provides a significant amount of fiber, vitamins, and minerals, as well as a low intake of saturated and trans fats, which could be described as an anti-inflammatory diet. However, from the 1960s to the present, the introduction of processed foods (especially processed meats), the regular consumption of eggs, and the use of dressings in Yaqui communities have had adverse repercussions on the nutritional quality of their traditional food systems [5,56]. On the other hand, historical records of the Seri diet, dating back more than one hundred years, describe it as one based on seasonal consumption of food that was obtained from the practice of a very rudimentary fishing using harpoons, complemented by activities of collecting fruits and seeds of plants from the desert and the sea, as well as hunting animals in the region, mainly characterized by the consumption of sea turtles, fish, aquatic birds, and terrestrial animals, as well as a moderate intake of vegetables such as pitaya, seagrass, and maguey [57,58]. More recent studies characterize the traditional Seri diet as highly consumed of low-fat cereals, wheat and corn tortillas, and legumes, in addition to fish and seafood [7]. This new dietary pattern is rich in monounsaturated and polyunsaturated fatty acids, fiber, vitamins, and minerals. However, foods from Westernized diets, such as beef, chicken, processed meat products, sugar-sweetened beverages, and sweet desserts, have been incorporated into their diets [7]. This diet is characterized by a high content of saturated fats and added sugars, as well as a low contribution of fiber, vitamins, and minerals, which can lead to a pro-inflammatory profile. In this context, the incorporation of meats and ultra-processed foods replaces traditional eating patterns, transforming a potentially anti-inflammatory diet into a pro-inflammatory one, which increases the risk of developing chronic non-communicable diseases, such as obesity.

The prevalence of obesity was high, similar to the national rates reported by the ENSANUT 2022 survey, although slightly lower among the IAS [3]. The same trend was observed for abdominal obesity [3]. As seen nationally, women in the IAS population were more prevalent than men [3]. According to Barquera et al. (2023), this gap may be attributed to socioeconomic and educational disparities, as well as nutritional deficiencies during early childhood [3].

Due to the study’s cross-sectional design, a limitation is that causality cannot be inferred; therefore, the observed association between the DII and both general and abdominal obesity in IAS cannot be considered causal. However, our findings are consistent with cohort studies that provide accumulating evidence regarding the directionality of this association [28,30,31,47]. Similarly, confirming this causal relationship through a cohort study conducted in this population, using biological inflammatory markers, would be important.

## 5. Conclusions

Higher scores of the DII were found to be associated with increased risk for both obesity indicators, BMI and WC, as well as both forms of obesity, in Indigenous adults from Sonora (IAS). An anti-inflammatory diet in this population, which helps reduce these indicators, is characterized by consuming foods rich in vitamins, minerals, fiber, and omega-3 fatty acids, as well as onions and chili peppers. Obesity, as well as abdominal obesity, constitutes a significant public health problem in Mexico, and IAS individuals are not exempt from this situation. The findings of this study not only suggest that promoting the adoption of anti-inflammatory diets could serve as an effective strategy for preventing these types of conditions but also provide a basis for designing culturally appropriate interventions and supporting the development of public health prevention strategies focused on promoting anti-inflammatory compound–rich foods that are already part of this population’s diet and recovering traditional foods from these communities that have anti-inflammatory potential. Ultimately, this work underscores the importance of generating contextualized evidence that incorporates the cultural, social, and dietary characteristics of Indigenous peoples, such as those of the Seri and Yaqui.

## Figures and Tables

**Table 1:** Comparison of anthropometric, sociodemographic, health history, and physical activity characteristics between the extreme tertiles of the DII (tertile 1 and 3) of Indigenous adults from Sonora.

	DII		
	T1 (−4.24, −1.42)	T3 (−0.16, 3.73)	*p*
**Anthropometrics**			
Weight (kg)	74.7 ± 15.9	77.3 ± 17.3	0.1171
BMI (kg/m^2^)	27.3 ± 5.3	28.6 ± 6.1	0.0280
WC (cm)	93.8 ± 12.8	95.9 ± 14.6	0.1416
**Sociodemographic**			
Age (years)	45.1 ± 13.6	39.4 ± 14.0	0.0001
Modernization index (score)	8.0 ± 3.1	8.6 ± 3.3	0.0608
Sex (women, %)	70.1	52.4	<0.0001
Indigenous language (yes, %)	93.5	76.2	<0.0001
Receives some form of support (yes, %)	78.1	71.3	0.1360
Beneficiary of the Seguro Popular program (yes, %)	69.5	65.9	0.4610
Marital status (%)			<0.0001
Single	22.5	16.8	
Married	57.2	39.4	
Consensual union	12.8	30.8	
Divorced or widowed	7.5	13.0	
Level of education (%)			0.0010
Primary or less	53.5	39.5	
Secondary	30.5	27.6	
High school or higher	16.0	32.9	
**Health history**			
Tobacco use (yes, %)	13.9	45.9	<0.0001
Parent with diabetes (yes, %)	50.2	32.9	0.0010
Previous diagnosis of hypertension (yes, %)	31.5	18.3	0.003
**Physical activity**			
Physical activity, types (h/week) *			
Sedentary	9.0 (3.2, 18.0)	12.9 (8.8, 23.1)	<0.0001
Light	28.6 (12.0, 53.5)	10.3 (0.9, 23.1)	0.0005
Moderate	15.1 (8.2, 27.7)	6.8 (1.8, 24.5)	<0.0001
Vigorous	0 (0, 1.8)	0 (0, 0.9)	0.0414

Numerical data are presented as mean ± standard deviation (* median and interquartile range), and categorical data are expressed as percentages. DII, Dietary Inflammatory Index; T1, tertile 1 of the DII; T3, tertile 3 of the DII; BMI, body mass index; WC, waist circumference; BP, blood pressure. The *p*-value corresponds to the comparison between sexes, calculated using *t*-tests, the Mann–Whitney test, and the Chi-square test.

**Table 2:** Analysis of parameter intake differences and corresponding DII scores between DII T1 and T3 groups among the Indigenous adults from Sonora.

Parameter		DII		
	T1 (DII: −4.24, −1.42)		T3 (DII: −0.16, 3.73)	
	Intake	Score	Intake	Score
β-carotene (µg/day) *^,†^	5049 (3549, 6589)	−0.24 ± 0.31	1239 (829, 1774)	0.45 ± 0.15
Vitamin D (µg/day) *^,†^	9.8 (6.4, 14.6)	−0.21 ± 0.31	2.5 (0.9, 3.6)	0.35 ± 0.17
Vitamin C (mg/day) *^,†^	167 (129, 216)	−0.23 ± 0.21	80 (56, 104)	0.19 ± 0.23
Magnesium (mg/day) *^,†^	467 (422, 519)	−0.34 ± 0.09	332 (290, 383)	−0.06 ± 0.18
Vitamin A (RAE/day) *^,†^	886 (695, 1146)	0.02 ± 0.19	386 (247, 520)	0.28 ± 0.09
Vitamin B6 (mg/day) *^,†^	2.8 (2.4, 3.1)	−0.32 ± 0.04	1.7 (1.4, 2.0)	−0.08 ± 0.15
Onion (g/day) *^,†^	32.4 (20.1, 57.4)	−0.01 ± 0.21	9.6 (6.8, 15.2)	0.22 ± 0.11
Zinc (mg/day) *^,†^	13.9 (12.5, 16.0)	−0.26 ± 0.07	11.2 (9.6, 12.3)	−0.09 ± 0.17
Omega-3 (g/day) *^,†^	2.0 ± 0.4	−0.26 ± 0.09	1.4 ± 0.3	−0.10 ± 0.09
Fiber (g/day) *^,†^	41.2 ± 13.3	−0.64 ± 0.06	34.8 ± 14.7	−0.49 ± 0.35
Vitamin E (mg/day) *^,†^	10.1 (8.8, 11.1)	−0.19 ± 0.22	9.1 (7.5, 10.7)	−0.06 ± 0.29
Selenium (µg/day) *^,†^	134 (117, 159)	−0.18 ± 0.03	87 (59, 110)	−0.06 ± 0.13
Pepper (g/day) *^,†^	21.7 (11.8, 46.8)	−0.07 ± 0.08	6.9 (3.5, 11.1)	0.03 ± 0.07
Niacin (mg/day) *^,†^	36.8 (32.5, 42.5)	−0.15 ± 0.07	31.8 (28.6, 34.9)	−0.08 ± 0.11
Energy (kcal/day) *	2995 ± 1034	0.10 ± 0.12	3378 ± 1204	0.13 ± 0.10
Omega-6 (g/day) *	16.9 (14.8, 18.8)	−0.09 ± 0.04	15.8 (13.2, 17.9)	−0.07 ± 0.04
Carbohydrates (g/day) *	416 ± 60	0.09 ± 0.03	457 ± 61	0.09 ± 0.2
Riboflavin (mg/day)	2.4 (2.2, 2.7)	−0.04 ± 0.02	2.5 (2.3, 2.8)	−0.04 ± 0.02
Iron (mg/day) *	27.7 (24.5, 30.7)	0.03 ± 0.01	34.5 (28.9, 40.0)	0.03 ± 0.01
MUFA (g/day) *	13.8 ± 5.2	0.01 ± 0.01	16.6 ± 6.2	0.01 ± 0.01
Trans fatty acids (g/day) *	0.6 ± 0.3	−0.12 ± 0.01	0.5 ± 0.3	−0.12 ± 0.01
Total fat (g/day)	101 ± 19	0.22 ± 0.10	100 ± 26	0.21 ± 0.09
Saturated fat(g/day)	31.4 ± 8.3	0.06 ± 0.19	31.3 ± 12.8	0.04 ± 0.16
PUFA (g/day)	10.2 ± 3.5	0.18 ± 0.15	10.7 ± 4.0	0.16 ± 0.18
Protein (g/day) *^,†^	138 ± 39	0.02 ± 0.00	94 ± 19	0.01 ± 0.01
Thiamin (mg/day) *^,†^	2.6 (2.2, 2.9)	−0.07 ± 0.03	3.2 (2.7, 3.8)	−0.08 ± 0.03
Alcohol (g/day) *^,†^	1.3 ± 5.7	0.27 ± 0.07	3.2 ± 12.0	0.24 ± 0.13
Cholesterol (mg/day) *^,†^	338 ± 166	0.03 ± 0.08	195 ± 119	−0.06 ± 0.08
Vitamin B12 (µg/day) *^,†^	6.9 (4.7, 9.5)	0.04 ± 0.06	2.3 (0.8, 3.4)	−0.07 ± 0.04
Folic acid (µg/day) *^,†^	297 (181, 380)	−0.01 ± 0.15	598 (485, 732)	−0.16 ± 0.09

DII, Dietary Inflammatory Index; T1, tertile 1; T3, tertile 3; MUFA, monounsaturated fatty acids; PUFA, polyunsaturated fatty acids. Intake expressed as mean (± standard deviation) or median (interquartile range). DII score expressed as mean (± standard deviation). * Difference between DII T1 and T3 groups expressed as parameters intakes (*p* < 0.05) by *t*-test or Mann–Whitney; ^†^ Difference between DII T1 and T3 groups expressed as DII scores (*p* < 0.05) by *t*-test.

**Table 3:** Multiple linear regression models of the association between BMI and WC with DII in Indigenous adults of Sonora.

Model	Hypothesis Variable	β	95% CI	*p*	*p* Trend
Dependent variable: BMI					
**Model 1**	Continuous DII (scores)	0.53	0.22, 0.85	0.001	
**Model 2**	Categorical DII				
	Tertile 1	Ref.	-	-	0.045
	Tertile 2	0.96	−0.16, 2.09	0.092	
	Tertile 3	1.86	0.72, 3.01	0.001	
Dependent variable: WC					
**Model 3**	Continuous DII (scores)	1.15	0.41, 1.87	0.002	
**Model 4**	Categorical DII				
	Tertile 1	Ref.	-	-	0.019
	Tertile 2	2.12	−0.47, 4.71	0.109	
	Tertile 3	3.81	1.16, 6.47	0.005	

BMI, body mass index (kg/m^2^); WC, waist circumference (cm); 95% CI, 95% confidence interval; DII, dietary inflammatory index; Ref., reference category. Models 1 and 2 were adjusted by sex, previous diagnosis of hypertension, modernization index, type of housing, and vigorous recreational physical activity. Models 3 and 4 were adjusted by sex, age, modernization index, previous diagnosis of hypertension, at least one parent with diabetes, and vigorous recreational physical activity.

**Table 4:** Multiple logistic regression models of the association between obesity and abdominal obesity with DII in Indigenous adults of Sonora.

Model	Hypothesis Variable	OR	95% CI	*p*	*p* Trend
Dependent variable: Obesity					
**Model 1**	Continuous DII (scores)	1.25	1.09, 1.43	0.001	
**Model 2**	Categorical DII				
	Tertile 1	Ref.	-	-	0.013
	Tertile 2	1.16	0.73, 1.88	0.497	
	Tertile 3	2.16	1.34, 3.45	0.002	
Dependent variable: Abdominal obesity					
**Model 3**	Continuous DII (scores)	1.20	1.01, 1.42	0.037	
**Model 4**	Categorical DII				
	Tertile 1	Ref.	-	-	0.760
	Tertile 2	2.26	1.24, 4.10	0.007	
	Tertile 3	1.69	0.95, 2.99	0.074	

OR, odds ratio; 95% CI, 95% confidence interval; DII, dietary inflammatory index; Ref., reference category. Models 1 and 2 were adjusted by sex, previous diagnosis of hypertension, modernization index, type of housing, and vigorous recreational physical activity. Models 3 and 4 were adjusted by sex, age, modernization index, previous diagnosis of hypertension, type of housing, sedentary occupational physical activity, and vigorous recreational physical activity.

## Data Availability

The collected and analyzed data during this study are available from the corresponding author upon reasonable request, subject to the intended use.

## Abbreviations

IAS	Indigenous adults from Sonora
DII	Dietary inflammatory index
BMI	Body Mass Index
WC	Waist Circumference
ENSANUT	Health and Nutrition Survey
NCDs	Non-communicable diseases
FFQ	Food frequency questionnaire
SD	Standard deviation
OR	Odds ratio
